# RYBP regulates *Pax6* during in vitro neural differentiation of mouse embryonic stem cells

**DOI:** 10.1038/s41598-022-06228-1

**Published:** 2022-02-11

**Authors:** Enikő Sutus, Surya Henry, Lili Adorján, Gergő Kovács, Melinda Katalin Pirity

**Affiliations:** 1 Institute of Genetics, Biological Research Centre, Szeged, Hungary; 2 Doctoral School in Biology, Faculty of Science and Informatics, University of Szeged, Szeged, Hungary; 3 Faculty of Science and Informatics, University of Szeged, Szeged, Hungary

**Keywords:** Developmental biology, Molecular biology, Neuroscience, Stem cells

## Abstract

We have previously reported that RING1 and YY1 binding protein (RYBP) is important for central nervous system development in mice and that *Rybp* null mutant (*Rybp*^−/−^) mouse embryonic stem (ES) cells form more progenitors and less terminally differentiated neural cells than the wild type cells in vitro. Accelerated progenitor formation coincided with a high level of *Pax6* expression in the *Rybp*^−/−^ neural cultures. Since *Pax6* is a retinoic acid (RA) inducible gene, we have analyzed whether altered RA signaling contributes to the accelerated progenitor formation and impaired differentiation ability of the *Rybp*^−/−^ cells. Results suggested that elevated *Pax6* expression was driven by the increased activity of the RA signaling pathway in the *Rybp*^−/−^ neural cultures. RYBP was able to repress *Pax6* through its *P1* promoter. The repression was further attenuated when RING1, a core member of ncPRC1s was also present. According to this, RYBP and PAX6 were rarely localized in the same wild type cells during in vitro neural differentiation. These results suggest polycomb dependent regulation of *Pax6* by RYBP during in vitro neural differentiation. Our results thus provide novel insights on the dynamic regulation of *Pax6* and RA signaling by RYBP during mouse neural development.

## Introduction

Signaling by the retinoic acid (RA) pathway is crucial for proper neural development both in vivo and *in vitro*^1^. During neural differentiation, RA signaling functions in a non-cell-autonomous manner depending upon the number of neighbouring cells that produce RA or express other signaling pathway members^2^. Prolonged RA induction and intercellular communication is a prerequisite of successful neural differentiation. However, RA can act cell-autonomously as well during vascular development of the brain^3^. In case of mouse embryonic stem (ES) cells RA induction facilitates neural differentiation in a concentration dependent manner and the differentiation of ES cells correlates with increased activity of Paired box 6 (*Pax6*), transcription factor, a gene important in central nervous system development^4^.

The transport of vitamin A1 metabolite RA into the nuclei of differentiating ectodermal cells is required for the initiation of neural differentiation. In the nucleus, RA binds to its own receptor complex (RAR-RXR heterodimers), which is bound to the RA-response elements (RAREs) present in the promoter of the target genes. After RA binding to the RAR-RXR heterodimer, the conformation of the RAR ligand-binding domain is altered, the co-repressors are released and co-activator complexes are recruited to facilitate the transcription of RA inducible genes such as *Pax6*^5^ (Fig S1).

PAX6 is an evolutionally highly conserved transcription factor governing eye and brain development^6^. Heterozygous loss-of-function mutations in *Pax6* cause aniridia in humans and the small eye phenotype in mice. Homozygous mutation of *Pax6* cause embryonic lethality at early stages in mouse model systems. In mouse, *Pax6* is expressed in a gradient throughout the developing cortex and is essential for corticogenesis. Region specific expression of *Pax6* at boundaries is highly important in order to establish anterior–posterior and dorsal–ventral brain axis. PAX6 is an important regulator of neuroprogenitor proliferation, neurogenesis, and formation of cortical layers. Furthermore, *Pax6* expression is necessary and sufficient for neuroectoderm specification in human but not in mouse ES cells^7^. The correct dosage of *Pax6* is critical for mouse neural development, especially for proper neural progenitor formation^8^ and high level of *Pax6* prevents terminal differentiation of neural progenitors.

Polycomb repressive complexes (PRCs) play a crucial role in regulating neurogenesis. The three distinct PRCs, namely Polycomb repressive complex 1 (PRC1), Polycomb repressive complex 2 (PRC2), and the non-canonical Polycomb repressive complexes 1 (ncPRC1s) work in coordination with each other to maintain the repressed state of key genes for developmental and fate decision making steps^9,10^. Both PRC1s and ncPRC1s contain Ring finger proteins (RING1, RNF2) and Polycomb group ring finger catalytic (PCGF) core, but in ncPRC1s, RING1 and YY1 binding protein (RYBP), or YY1 associated factor 2 (YAF2), replaces the Chromobox (CBX) and Polyhomeotic (PHC) subunits found in cPRC1s^11–15^. RYBP and YAF2 proteins are mutually exclusive regulatory subunits of ncPRC1s. There are at least six polymorphic ncPRC1 complexes (ncPRC1.1–1.6) described so far based on the presence of relevant PCGF subunits (PCGF1-6). NcPRC1s regulate cell lineage specifications and differentiation by suppressing the activation of alternative cell fate choices^16,17^. Mutations of the ncPRC1s resulted in various neural defects: incomplete neural tube closure, exencephaly, premature neural differentiation and altered cell-cycle processes in neural precursors, which can often lead to neurodevelopmental disorders, intellectual disability and autism^17–21^.

We have previously reported that *Rybp* is essential for mouse embryonic development and decreased dosage of *Rybp* caused exencephaly in mice^22^. Furthermore, ES cells lacking functional *Rybp* formed less matured neural cell types from existing progenitors in vitro than the wild type cells and exhibited altered expression of a series of key neural marker genes including *Pax6*^23^. RYBP, is Zinc finger protein, which can recognize nucleosomes containing H2AK119Ub marks, It can bind to ubiquitylated proteins and as a part of ncPRC1s, RYBP facilitates H2A ubiquitylation of targets^15^. Besides the Zinc finger motif of RYBP, it also contains intrinsically disordered stretches conferring multiple tertiary structure of the protein. Partially due to these characteristics, RYBP interacts with a versatile set of proteins with diverse functions (moonlighting function). RYBP was first described as interactor of the mammalian polycomb group proteins and as an epigenetic regulator involved in chromatin compaction^24^. Besides its role in neural development, the function of RYBP has been investigated during haematopoetic, cardiac and germ cell development and also in tumour formation^25–28^, RYBP was also described as a pro-apoptotic protein and interactor of procaspase-8 and DEDD via a non-epistatic mechanism^29^. However multiple biological functions of RYBP has been described, the precise molecular mechanism of specific functions are not well established and target genes of RYBP actions are not well defined.

In the current study, we have investigated the molecular mechanisms that might be responsible for the increased progenitor formation of the *Rybp*^−/−^ ES cells. We examined whether the modulation of the RA signaling pathway members could transmit the effects of RYBP and whether this can be the reason of the elevated *Pax6* level in the *Rybp*^−/−^ neural cultures. We used a stem cell based in vitro differentiation system and compared the effect of RA on the expression of *Pax6* and other members of the RA signaling pathway in the presence and absence of *Rybp* during in vitro neural differentiation. Since RYBP exerts its functions partially as a core member of the ncPRC1s, we investigated whether the function of RYBP is mediated via ncPRC1s to regulate *Pax6* gene expression. Here we report that in the lack of *Rybp,* the expression of the RA signaling pathway members and their target genes, such as *Pax6*, were upregulated during neural differentiation. By using luciferase reporter assays, we revealed that RYBP is able to directly repress the *P1* promoter of the *Pax6* gene and that this repression was enhanced in the presence of RING1. These findings suggest that RYBP regulates *Pax6* expression partially by governing the expression of several RA pathway genes and by repressing the activity of *Pax6 P1* promoter via ncPRC1s.

## Results

### The expression of RA pathway members is altered in the lack of *Rybp* in ES cells

In order to investigate whether the lack of *Rybp* altered RA signaling related gene expression, we analyzed our previously published transcriptome dataset from wild type and *Rybp*^−/−^ ES cells^30^ (GEO acc. GSE151349) (Materials and Methods) focusing on the expression of genes related to RA signaling.

**Figure 1:**
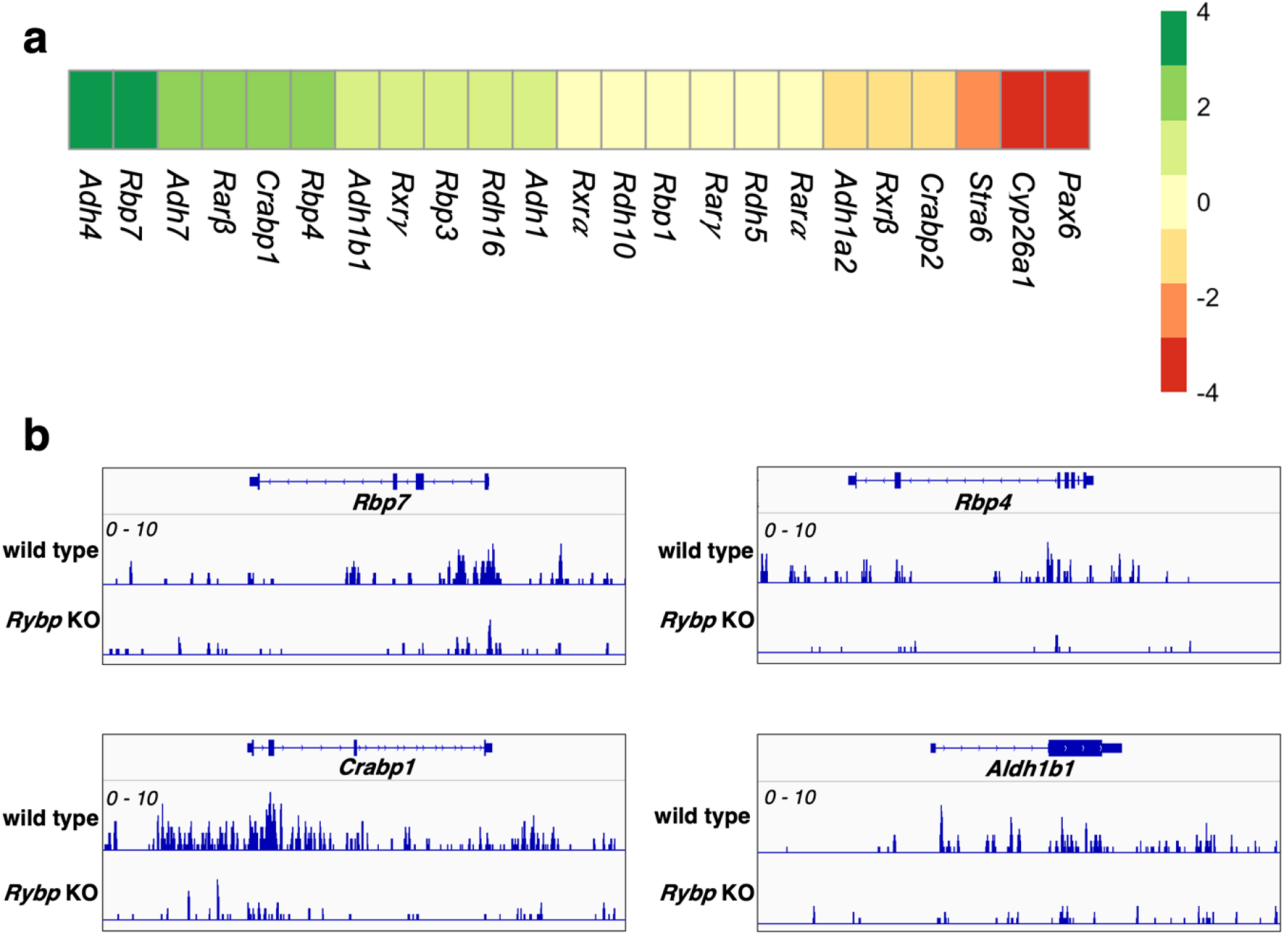
Gene expression changes of RA signaling pathway members in wild type and *Rybp*^−/−^ ES cells. (**a**) Heatmap of RA signaling related gene expression changes comparing *Rybp*^−/−^ ES cells to wild type cells revealed altered expression of several RA pathway member genes. Green and red indicate upregulation (log^2^ fold change ≥ 1.5) and downregulation (log^2^ fold change ≤  − 1.5) respectively and yellow indicates genes that did not display significant expression changes. Heatmaps were generated using pheatmap (R package version 1.0.12) in Rstudio 1.3.959 ( http://www.rstudio.com/) (**b**) ChIP-seq analysis of H2AK119ub1 marks in wild type and *Rybp*^−/−^ ES cells revealed loss of H2AK119ub1 at the genomic locus of RA pathway members. Data range 1–10.

First, we collected RA pathway members and some designated RA inducible genes^31,32^ from our transcriptome (23 hits) and visualized their log^2^ fold change values on heatmap (Fig. 1a). From the 23 hits, 10 genes were deregulated (− 1.5 ≥ log^2^ fold change ≥ 1.5). In detail, there were 7 genes upregulated in *Rybp*^−/−^ ES cells including Alcohol dehydrogenase 4 (*Adh4*), Retinol binding protein 7 (*Rbp7*), Alcohol dehydrogenase 7 (*Adh7*), Retinoic acid receptor beta (*Rarβ*), Cellular retinoic acid binding protein (*Crabp1*), Retinol binding protein 4 (*Rbp4*), Aldehyde dehydrogenase 1 family member B1 (*Aldh1b1*) and 3 genes were downregulated including *Pax6*, Cytochrome P450 Family 26 Subfamily A Member 1 (*Cyp26a1*) and Signaling receptor and transporter of retinol (*Stra6*). From the 10 deregulated genes, 5 genes namely *Rarβ*, *Crabp1*, *Stra6*, *Cyp26a1* and *Pax6* are RA inducible genes. This showed that the absence of *Rybp* affected the expression of the RA pathway members and the RA inducible genes.

Next, we checked the H2AK119ub1—a major repression mark in the wild type and *Rybp* KO ES cells using previously published ChIP-seq datasets^33^ (SRA acc. SRP247114) at the genomic loci of those 7 genes which were upregulated in *Rybp*^−/−^ ES cells (Fig. 1a). We found loss of H2AK119ub1 mark in *Rybp*^−/−^ cells at the genomic loci of 4 genes namely *Rbp7*, *Crabp1*, *Rbp4*, *Aldh1b1* (Fig. 1b). The loss of H2AK119ub1 marks in the genomic loci of specific RA pathway members in the *Rybp*^−/−^ cells indicated that RYBP might regulate these genes in a polycomb dependent way. These results, and the elevated *Pax6* gene expression in the *Rybp*^−/−^ neural cultures^23^ encouraged us to further analyze the detailed expression changes of RA pathway members during in vitro neural differentiation.

### Expression of several RA signaling pathway members are amplified during in vitro neural differentiation of the *Rybp*^−/−^*stem cells*

Since *Pax6* is robustly activated by RA signaling during neural development, we examined whether the expression of RA signaling pathway members is attenuated after all-trans retinoic acid (atRA, also known as tretinoin) treatment and whether the *Rybp*^−/−^ neural cultures express more *Pax6* than the wild type cells. To answer this question, wild type and *Rybp*^−/−^ ES cells were differentiated to neural lineages in vitro for 14 days (d) by using the modified protocol of Bibel et al.^34^ (Materials and methods, in vitro neural differentiation). Samples were collected for analysis at day 0 (d0; representing ES cell stage), day 3 (d3; representing embryoid body (EB) stage before atRA treatment), day 7 (d7; representing EB stage after atRA treatment), day 10 (d10; representing an early stage of neural differentiation) and day14 (d14; representing the late stage of neural differentiation) (Fig S2a).

For monitoring the mRNA expression changes, qRT-PCR (Materials and methods, Quantitative real-time PCR (qRT-PCR)) was performed using primers specific to *Stra6,* Retinol binding protein 1 (*Rbp1*, also known as *Crbp1*)*,* Retinol binding protein 2 (*Rbp2*, also known as *Crbp2*)*,* Retinol dehydrogenase 10 (*Rdh10*) and Retinol dehydrogenase 14 (*Rdh14*)*,* Aldehyde dehydrogenase 1 family member A1 (*Aldh1a1*, also known as *Raldh1*) and A2 (*Aldh1a2*, also known as *Raldh2*)*, Crabp1,* Cellular retinoic acid binding protein 2 (*Crabp2*)*,* Retinoic acid receptor alpha (*Rarα*), *Rarβ*, Retinoid X receptor alpha (*Rxrα*), Retinoid X receptor beta (*Rxrβ*) and Cytochrome P450 Family 26 Subfamily B Member 1 (*Cyp26b1*) were monitored by qRT-PCR (primer details in Table S1).

**Figure 2:**
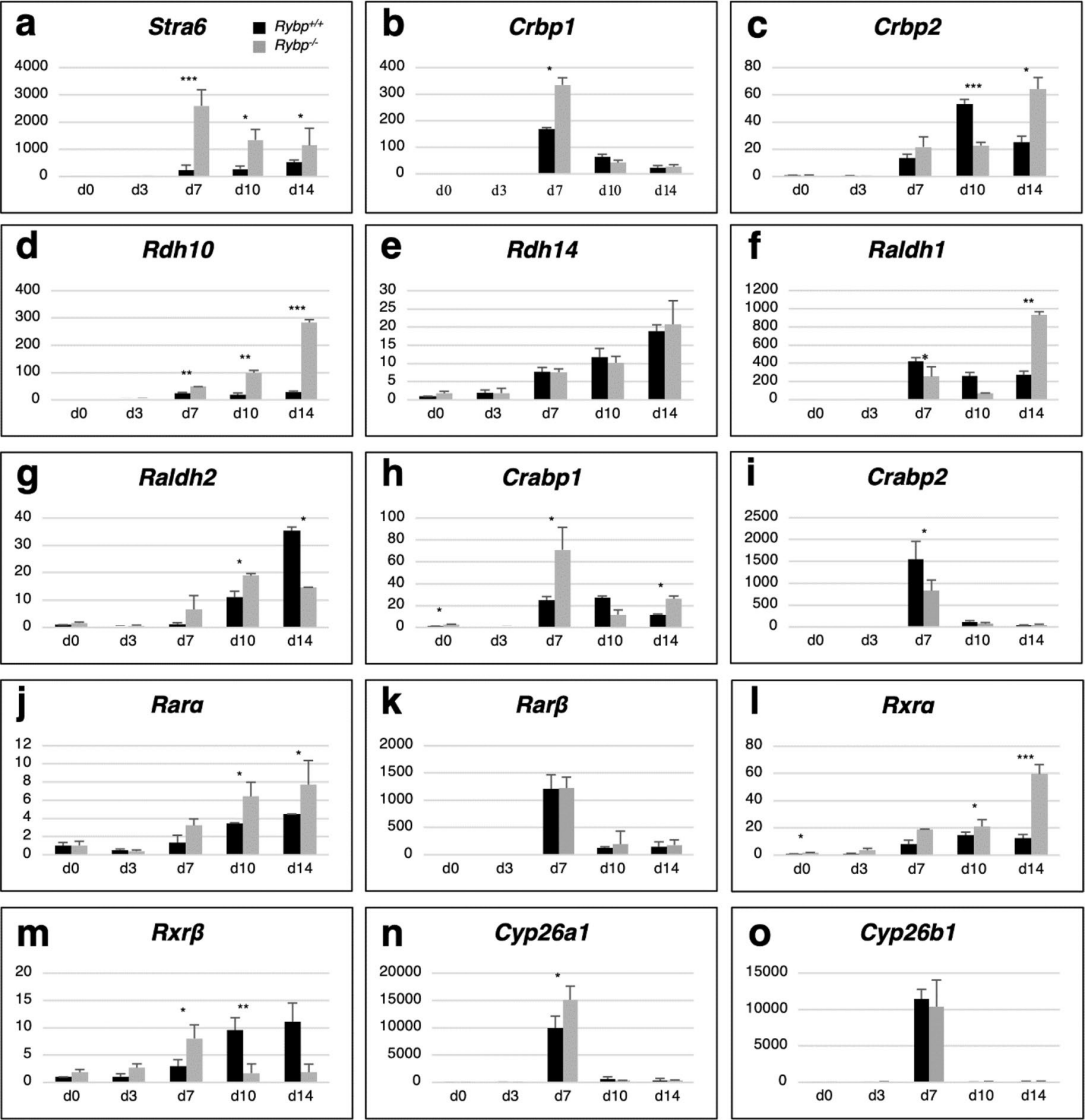
Members of the RA signaling pathway exhibit elevated expression level in the absence of *Rybp*. Relative gene expression changes of membrane receptor (**a**) *Stra6*; intracellular retinol binding complex members (**b**) *Rbp1*, (**c**) *Rbp2*; and retinoid converting enzymes (**d**) *Rdh10*, (**f**) *Aldh1a1*, (**g**) *Aldh1a2* by qRT-PCR showed altered expression in *Rybp*^−/−^ neural cultures in comparison to wild type, while there was no significant difference in (**e**) *Rdh14* expression in the two cell lines. Relative gene expression analysis of intracellular RA binding complex members (**h**) *Crabp1*, (**i**) *Crabp2*; nuclear RA receptor complex members (**j**) *Rarα*, (**l**) *Rxrα*, (**m**) *Rxrβ* and RA degrading enzyme (**n**) *Cyp26a1* by qRT-PCR showed altered expression in the *Rybp*^−/−^ neural cultures in comparison to the wild type but there was no difference in (**k**) *Rarβ* and (**o**) *Cyp26b1* expression between the two cell lines. The expression of the indicated markers was normalized to *Hprt* level. Means are standard deviation ± SD. Values of *p* < 0.05 were accepted as significant (*p < 0.05; **p < 0.01; ***p < 0.001). Statistical method: *t* test type 3, n = 3.

As expected, *Stra6* was robustly induced by atRA in both cell lines at every examined time point (d7, d10, d14). Furthermore, *Stra6* was induced at a higher extent in the *Rybp*^−/−^ cells compared to the wild type cells (Fig. 2a). The expression of both *Rbp1* (Fig. 2b) and *Rbp2* (Fig. 2c) increased after atRA treatment in the wild type cells, and the mRNA levels were significantly higher in the *Rybp*^−/−^ cells. We also assessed the expression changes of the retinol metabolizing *Rdh10* and *Rdh14*, the retinal metabolizing *Aldh1a1* and *Aldh1a2* dehydrogenase enzymes. The expression of these genes was strongly enhanced after atRA treatment (d7) and *Rdh10* expression reached the highest level in the *Rybp*^−/−^ cells by the end of differentiation (d10 and d14) (Fig. 2d). There were no significant differences in *Rdh14* expression between the two cell lines, its level gradually increased after atRA treatment until the endpoint of in vitro differentiation (Fig. 2e). *Aldh1a1* expression kinetics was different in the two cell lines: it peaked right after atRA induction (d7) in the wild type cells, and it was most extensively expressed by the end of differentiation (d14) in the *Rybp*^−/−^ cells and the induction was 2.5 times higher compared to the wild type at this time point (Fig. 2f). In contrast, *Aldh1a2* gene expression was higher at d7 and d10 in the absence of *Rybp* in comparison to the wild type (Fig. 2g). The expression changes of the cytoplasmic RA binding complex members *Crabp1* and *Crabp2* peaked after atRA treatment (day 7). At d7 and d14 *Crabp1* expression was also more pronounced (Fig. 2h) than *Crabp2* (Fig. 2i) in the *Rybp*^−/−^ mutant.

Furthermore, gene expression analysis revealed upregulation of the nuclear RA binding *Rar/Rxr* complex members. We found that *Rarα*, exhibited persistently higher expression peaks in the *Rybp*^−/−^ cells compared to the wild type at d10 and d14 (Fig. 2j). The two cell lines did not show any difference in the *Rarβ* expression (Fig. 2k). The expression of both *Rxr* members showed a gradually increasing level in the *Rybp*^−/−^ cells. *Rxrα* level was higher at all examined time points in the *Rybp*^−/−^ cell line and its expression kinetics did not decline by the end of differentiation as it did in the case of the wild type (Fig. 2l). *Rxrβ* level was also higher in the *Rybp*^−/−^ cells, but compared to the *Rxrα,* it showed higher mRNA level at d0, d3 and the highest at d7 (after atRA treatment) (Fig. 2m). The mRNA level of RA degrading enzyme *Cyp26a1* was strongly induced by atRA treatment (d7), and the level of induction was more pronounced in the *Rybp*^−/−^ compared to the wild type (Fig. 2n). There were no differences in *Cyp26b1* expression in the two cell lines (Fig. 2o).

These results showed that the expression of all examined genes, which are responsible for retinoid binding and metabolism in the cytoplasm (*Rbp1*, *Rbp2, Rdh10, Aldh1a2* and *Crabp1)* was higher in the *Rybp*^−/−^ cells from neuroectoderm formation (after day 4) until the last day (d14) of in vitro neural differentiation. Notably, the expression level of RA inducible genes, such as *Stra6* and *Cyp26a1*, were significantly higher after atRA treatment (d7) in the absence of *Rybp*. Taken together, these data suggested that the lack of *Rybp* led to an amplified RA response in the cells during in vitro neural differentiation.

### PAX6 is upregulated in the *Rybp*^−/−^ neural cultures in response to RA signaling

**Figure 3:**
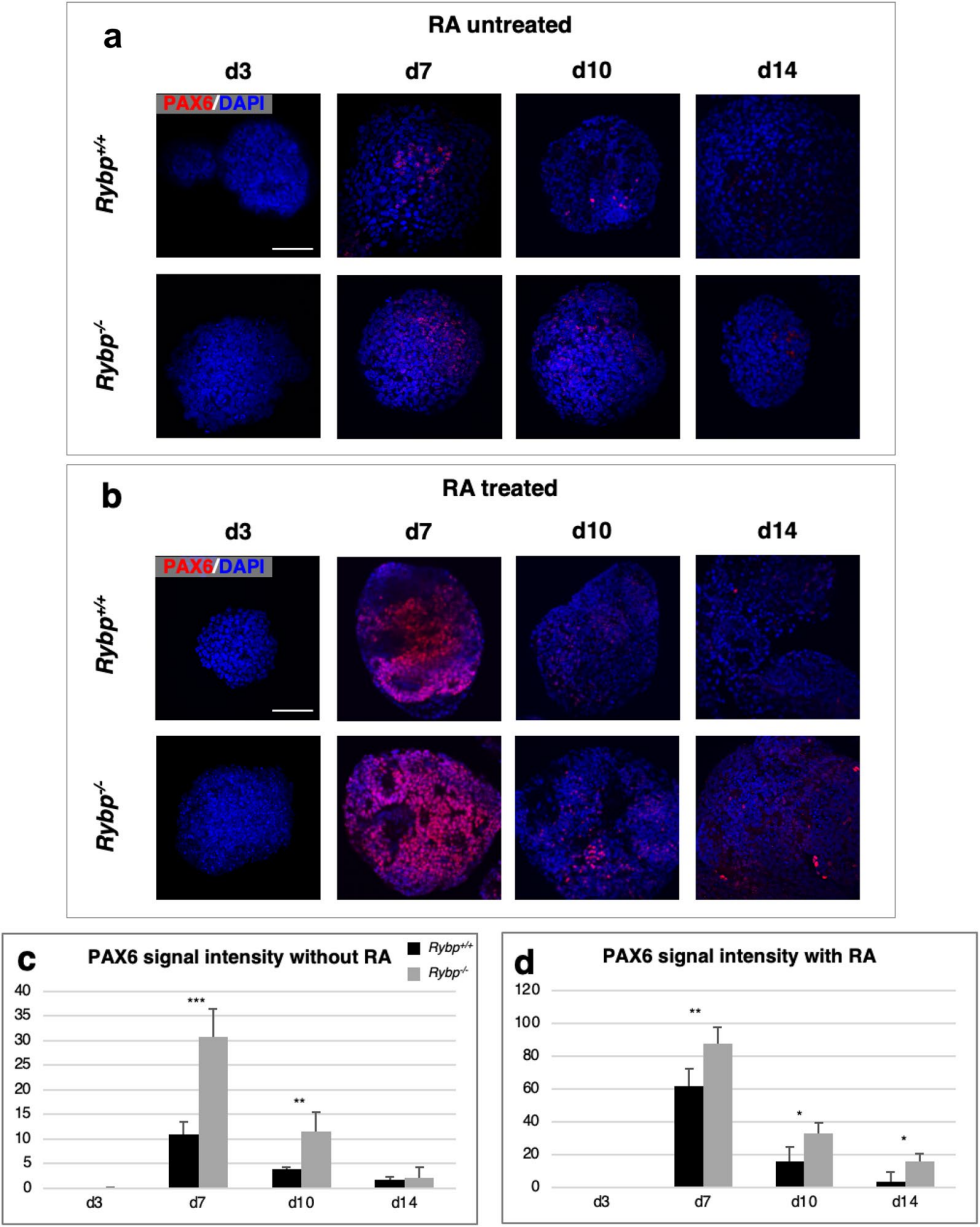
PAX6 is strongly upregulated in the lack of *Rybp* (*Rybp*^−/−^) during neural differentiation of ES cells. Immunocytochemical analysis of PAX6 protein level in EBs in the (**a**) absence and (**b**) presence of atRA revealed increased PAX6 signals in the *Rybp*^−/−^ cells compared to the wild type cells. Objective: 40 × . Scale bar: 80 μm. (**c**,**d**) ImageJ quantification of PAX6 protein level in the wild type and *Rybp*^−/−^ cells during the in vitro neural differentiation in the absence (**c**) and presence (**d**) of atRA. Means are standard deviation ± SD. Values of *p* < 0.05 were accepted as significant (*p < 0.05; **p < 0.01; ***p < 0.001). Statistical method: *t* test type 3, n = 3.

Next, we examined whether the elevated level of *Pax6* indeed depends on the atRA treatment. First, we investigated whether PAX6 is profoundly present in the *Rybp*^−/−^ cells even without atRA treatment (Fig. 3a). In this experiment, both the wild type and the *Rybp*^−/−^ ES cells were grown in suspension culture for promoting EB formation for 14 days with and without atRA (Materials and methods, Embryoid body formation and atRA treatment). We added atRA on d4 to the cultures in case of the atRA treated samples, to facilitate neuroectoderm formation (Fig S2b). Samples were collected at different time points (d3, d7, d10 and d14) and were proceeded for ICC (Materials and methods, Immunocytochemistry (ICC)) to compare the PAX6 protein level in the wild type and the *Rybp*^−/−^ lines (Fig. 3c,d).

PAX6 protein was detected as early as d3 both in the wild type and the *Rybp*^−/−^ EBs at a very low level (Fig. 3a,b). As expected, at later time points (d7, d10 and d14) the staining intensity of PAX6 was stronger in the atRA treated EBs (Fig. 3b–d) compared to the untreated EBs (Fig. 3a,c,d) in both cell lines. Results were further confirmed by quantification of the PAX6 protein signals by ImageJ (Fig. 3c,d).

These results showed that even without atRA treatment PAX6 was present in an excess in the absence of *Rybp* in comparison to the wild type EBs, and this difference was more pronounced after atRA treatment.

### The localization of RYBP and PAX6 proteins dynamically change during in vitro neural differentiation

**Figure 4:**
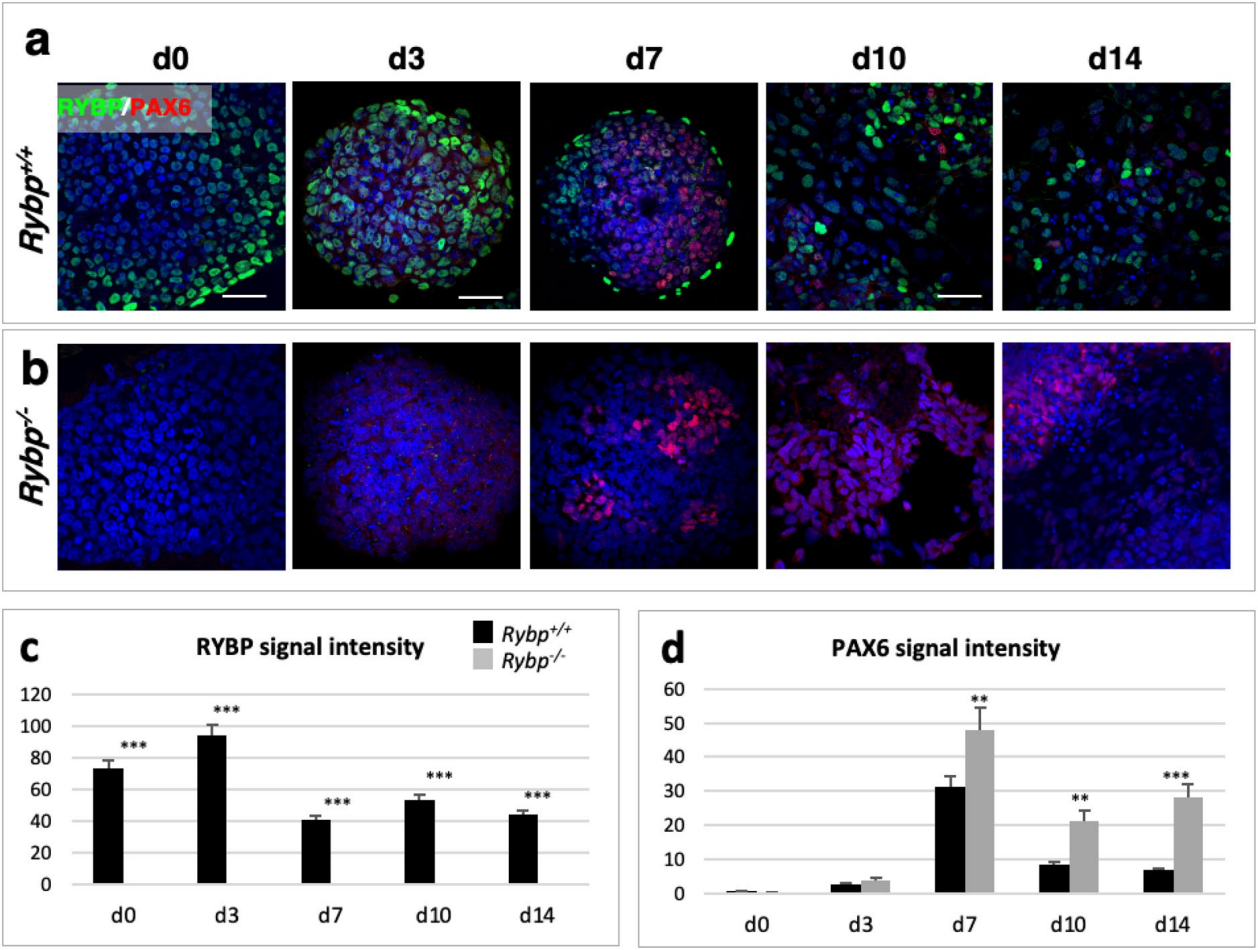
Immunocytochemical localization of RYBP and PAX6. Immunocytochemical analysis of RYBP and PAX6 protein level in (**a**) *Rybp*^+*/*+^ and (**b**) *Rybp*^−/−^ cells during in vitro neural differentiation. Objective: d0, d10 and d14: 60x; d3 and d7: 40x. Scale bar: d0, d10 and d14: 100 μm; d3 and d7: 80 μm. (**c**,**d**) ImageJ quantification of (**c**) RYBP and (**d**) PAX6 protein level in the wild type and *Rybp*^−/−^ cells during in vitro neural differentiation. Means are standard deviation ± SD. Values of *p* < 0.05 were accepted as significant (*p < 0.05; **p < 0.01; ***p < 0.001). Statistical method: *t* test type 3, n = 3.

To reveal the spatiotemporal distribution of PAX6 and RYBP in comparison to each other, we first subjected the wild type ES cells to in vitro neural differentiation (Materials and methods, in vitro neural differentiation) and analyzed the localization of the two proteins at d0, 3, 7, 10, and 14 of in vitro neural differentiation by ICC (Materials and methods, Immunocytochemistry (ICC)). Samples were examined using an Olympus LSM confocal microscope (Fig. 4a,b). ImageJ software was used to quantify the obtained fluorescent signals (Fig. 4c,d).

The most RYBP positive cells could be observed in ES (d0) cell stage and at early differentiation stages (d3) (Fig. 4a). After the atRA treatment, the amount of RYBP decreased (d7) and it appeared mostly in the outer layer of the EBs, where endoderm forms. (Fig. 4a). By d10 and 14, less sparsely spread RYBP positive signals were visible in the neural cultures (Fig. 4a). As expected, the most PAX6 positive signals appeared in cells at d7, after atRA treatment, and very few PAX6 signals were present at d10 and 14 in the wild type cultures (Fig. 4a). By subjecting *Rybp*^−/−^ cells to in vitro neural differentiation as well, PAX6 appeared to be localized abundantly at d7, d10 and d14 in comparison to the wild type cells. (Fig. 4b,d). As expected, there was no detectable RYBP signal in the *Rybp*^−/−^ cells at any time point of in vitro neural differentiation. Notably, the amount of PAX6 was higher after the atRA treatment (d7) in the *Rybp*^−/−^ cells and PAX6 did not decrease by the end of differentiation (d10 and d14) (Fig. 4d).

These results indicated that in differentiating cells, where there is no RYBP, more PAX6 protein appeared and that the two proteins are not or rarely co-localized.

### RYBP and PAX6 are co-expressed at progenitor stage of in vitro neural differentiation

**Figure 5:**
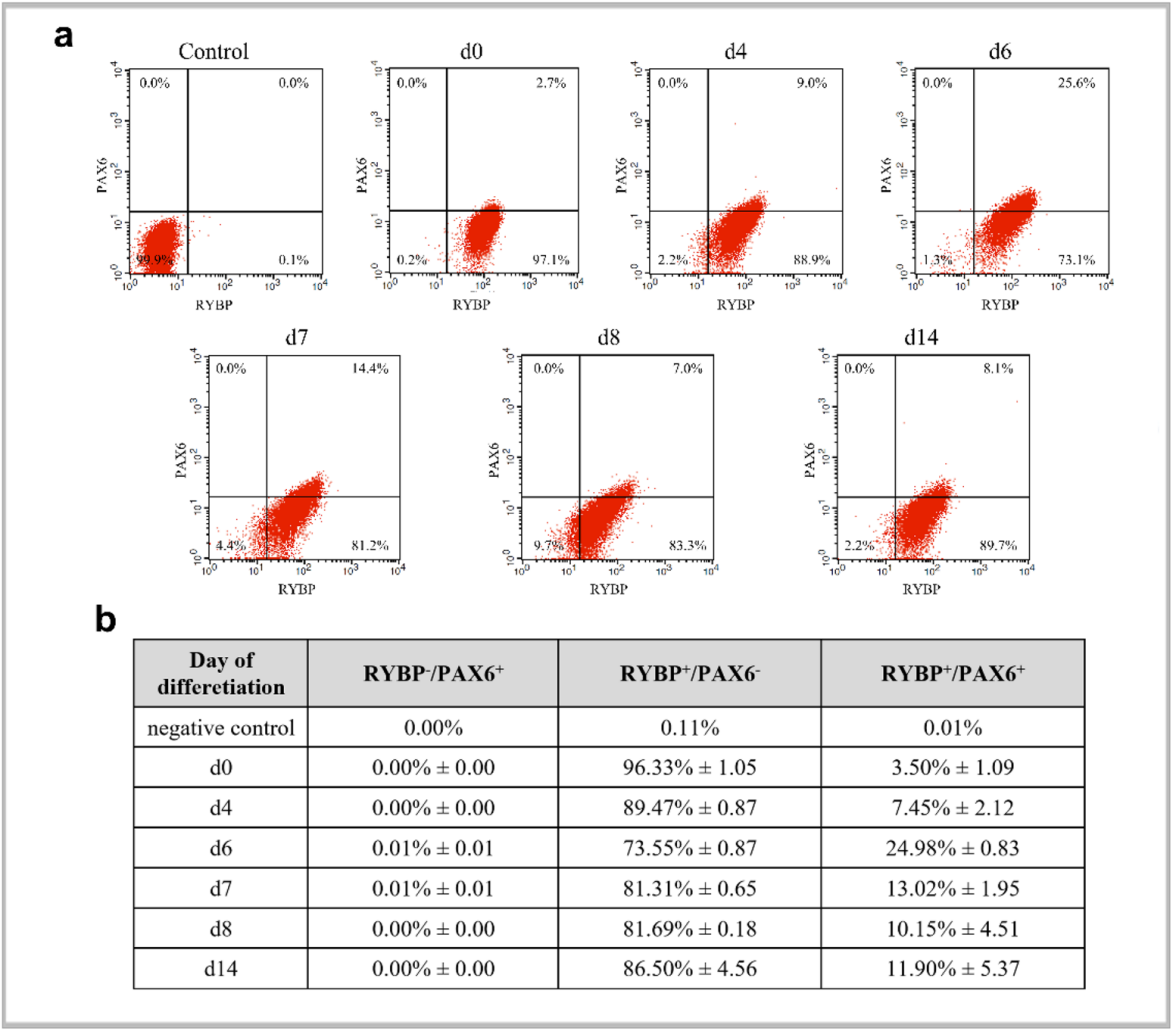
RYBP and PAX6 are co-expressed in cells during in vitro neural differentiation. The wild type ES cells were induced for differentiation with atRA for various periods of time (d4, d6, d7, d8 and d14) or left unstimulated (d0). The cells were stained with mouse monoclonal PAX6 and rabbit polyclonal RYBP/DEDAF primary antibodies followed with Alexa Fluor 647 labelled Donkey anti Mouse IgG (H + L) and Alexa Fluor 488 labelled Donkey anti Rabbit IgG (H + L), respectively then analyzed with cytofluorimetry. Control means, that the first antibodies were omitted and both secondary antibodies were added to the samples (**a**,**b**). Representative FACS plots of two independent experiments is shown at (**a**). Data are represented as means me of two biological repeats (n = 2). (**b**) Percentage and standard deviation ( ±) of control, RYBP^−^/PAX6^+^, RYBP^+^/PAX6^−^ and RYBP^+^/PAX6^+^ cells during in vitro neural differentiation of the wild type ES cells. Percentages were calculated with BD CellQuest Pro Version 6.0 software.

Previous experiments suggested that RYBP and PAX6 expressed complementary in most of the cells during neural differentiation. Therefore, co-expression was analyzed in an in vitro neural differentiation model system by FACS analysis. Wild type ES cells were exposed to atRA (Materials and methods, in vitro neural differentiation) for different times (d4, 6, 7, 8 and 14), or left unstimulated and subsequently samples were single (data not shown) or double labeled (Fig. 5) with RYBP and PAX6 antibodies then analyzed with flow cytometry analysis (Materials and methods, Flow Cytometry).

The analyses showed that the ratio of RYBP^+^/PAX6^+^ double positive cells increased up to 6th day of atRA treatment (Fig. 5b) then decreased after d7 and remained at similar level till d14 of differentiation. (Fig. 5b). These results showed that the most RYBP^+^/PAX6^+^ cells appeared at the progenitor cell stage and that the number of RYBP^+^/PAX6^+^ cells decreased by differentiation.

### RYBP represses *Pax6* expression through its *P1* promoter

**Figure 6:**
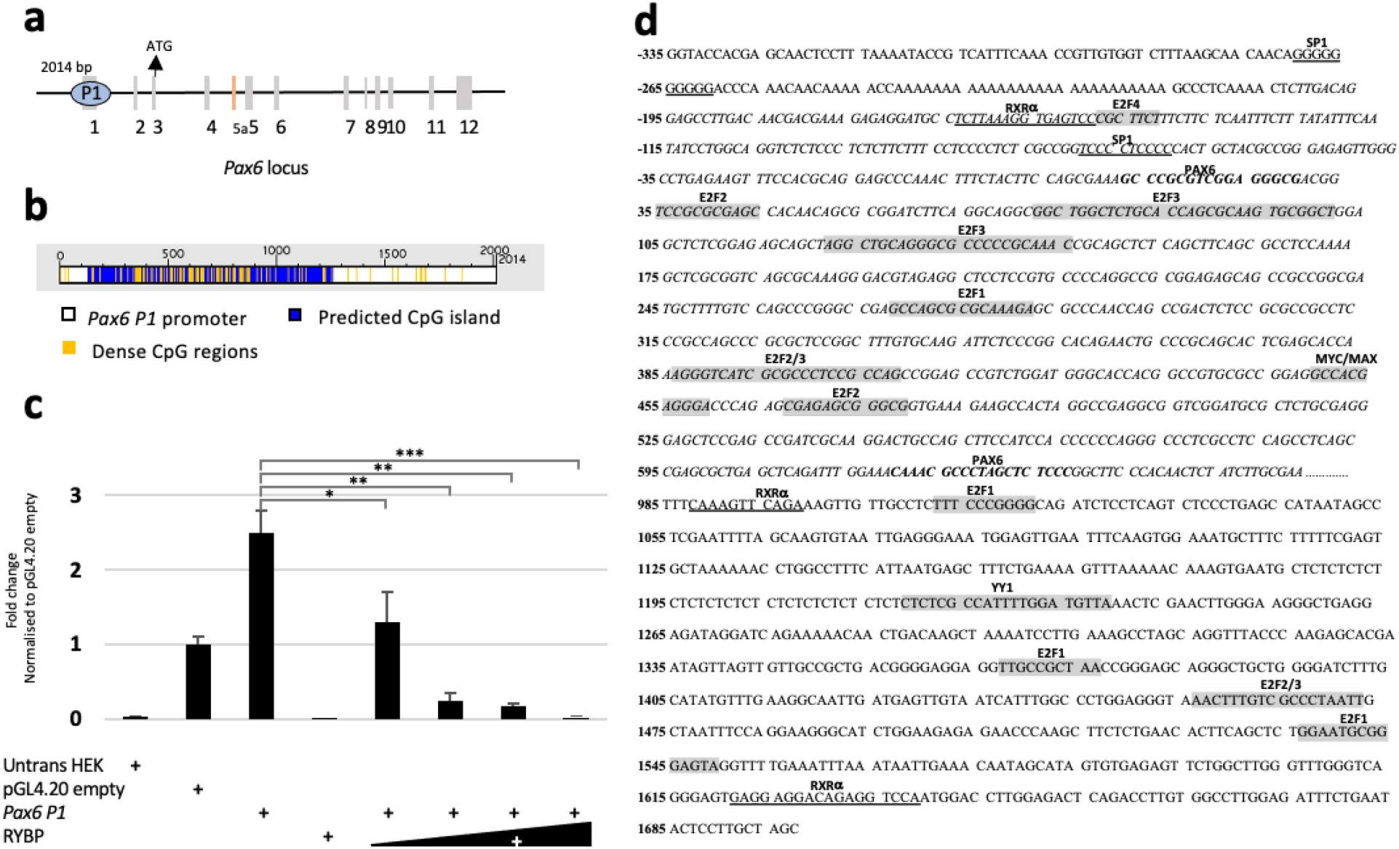
RYBP represses *Pax6* gene expression through the *Pax6 P1* promoter. (**a**) The *Pax6* genomic locus showing the position of the 2014 bp *P1* promoter used in this study. (**b**) CpG island prediction at the *Pax6 P1* promoter was performed in DBCAT (http://dbcat.cgm.ntu.edu.tw). The predicted CpG island is represented in blue and the denser CpG regions are displayed in yellow. (**c**) Luciferase assays were performed in HEK293 cells transiently transfected with mouse *Pax6 P1* promoter reporter (pGL4.20 *Pax6 P1*) or with empty vector (pGL4.20) and RYBP expression plasmid in increasing dosages (1 µg, 5 µg, 10 µg and 20 µg). Values are expressed as fold changes of luciferase activity normalized to the empty vector. The presented values are averages of three independent experiments. Means are standard deviation ± SD. Values of *p* < 0.05 were accepted as significant (*p < 0.05; **p < 0.01; ***p < 0.001, n = 3). Statistical method: T test type 3. (**d**) *Pax6 P1* promoter highlighting the position of key transcription factor binding sites and CpG islands. CpG island is represented in italics. The transcription factor binding sites of RYBP interacting proteins are highlighted in grey. PAX6 self-binding sites are represented in bold, RXRα and SP1 binding sites are underlined. *Untrans HEK* Untransfected HEK293 cells, *SP1* Trans-acting transcription factor 1, *E2F* E2 transcription factor, *MYC*/*MAX* Myelocytomatosis oncogene/MYC associated factor X, *YY1* Yin-yang-1 transcription factor.

Since previous results suggested that RYBP might repress *Pax6* gene expression at developmental stage specific manner, we next examined whether RYBP can indeed regulate *Pax6* expression through its promoter using the luciferase reporter assay system. For this assay, we utilized a 2014 bp long *P1* promoter region (-335 to 1698 from TSS) (Materials and methods, Cloning of *Pax6 P1* promoter) of *Pax6* that encompassed the entire exon 1 (Fig. 6a) and included a predicted 1127 bp long CpG island that covers 55.9% of the promoter region (Fig. 6b).

To evaluate the regulatory potential of RYBP at the *Pax6 P1* promoter, HEK293 cells were co-transfected with the *Pax6 P1* promoter construct in combination with different dosages of the *Rybp* cDNA containing construct^35^ (1 µg, 5 µg, 10 µg and 20 µg) (Materials and Methods, Luciferase reporter assay). 40 h after transfection, the protein cell lysates were harvested, processed and luciferase signals were measured. Our results showed over 2-folds repression of the *Pax6 P1* promoter by RYBP in comparison to the control cell lysates (Fig. 6c). The fold decrease was proportional to RYBP dosage, and the luciferase levels reached almost the base level when 20 µg of RYBP was applied (Fig. 6c). Since RYBP influenced a sharp decrease in the luciferase levels of the *Pax6 P1* promoter, we performed transcription factor binding site (TFBS) analysis of the *Pax6 P1* promoter using the TRANSFAC tool (https://genexplain.com/transfac). This revealed various known binding partners of RYBP that could synergistically function in regulating the expression of *Pax6*^24^ (Fig. 6d). This included several E2 transcription factor (E2F) binding sites (E2F1, E2F2, E2F3 and E2F4), a Yin-yang-1 transcription factor (YY1) binding site and a Myelocytomatosis oncogene/MYC associated factor X (MYC/MAX) consensus sites (Fig. 6d). The promoter region also contained RXR*α* and PAX6 binding sites. This is in accordance with previously published data stating that RA signaling plays a crucial role in the regulation of *Pax6*^36,37^ (Fig. 6d). These all together suggested that RYBP could potentially recruit repressor complexes via its binding partners and could repress *Pax6* gene expression through its *P1* promoter.

### RYBP represses *Pax6* gene expression in a polycomb dependent manner

**Figure 7:**
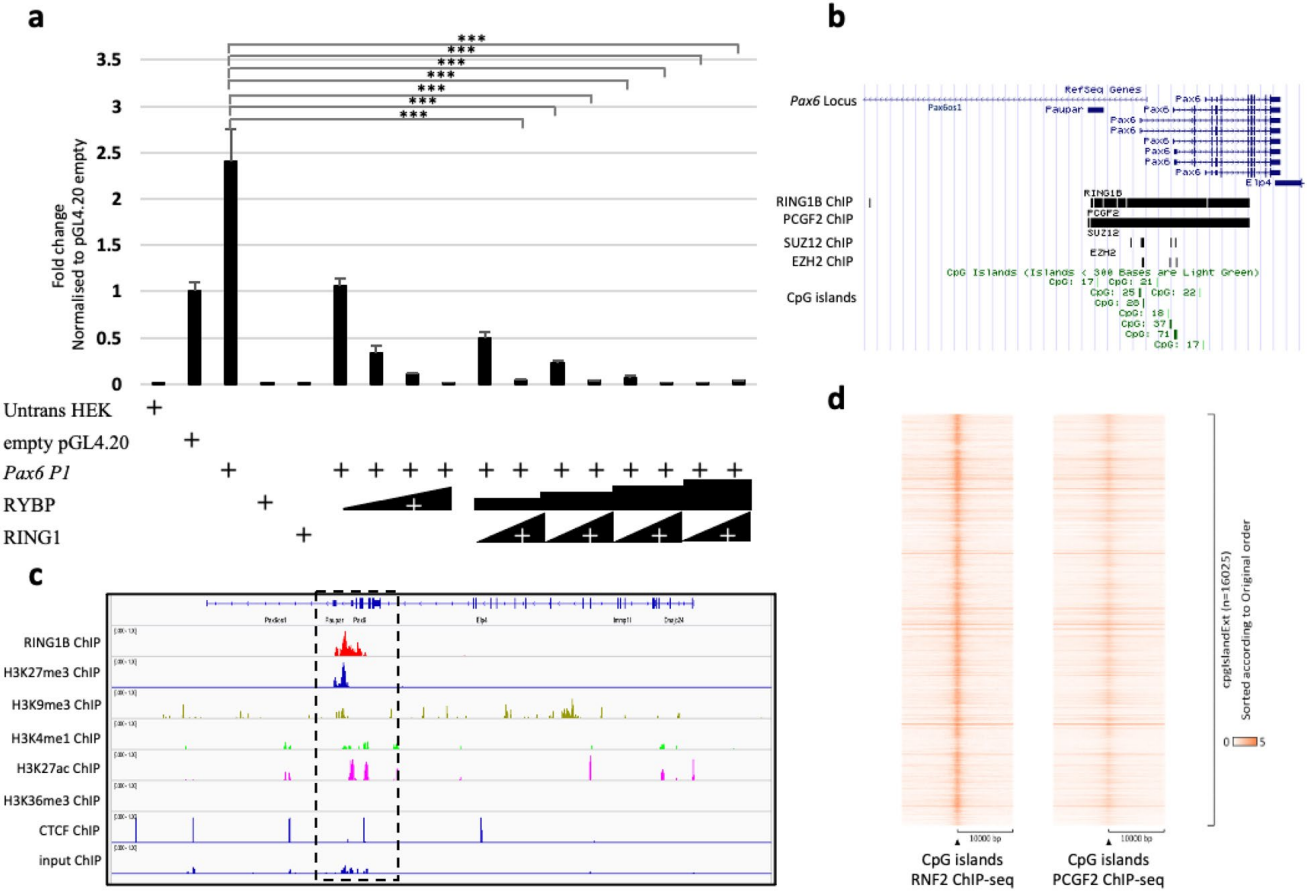
RYBP represses *Pax6* gene expression in a polycomb dependent way. (**a**) Luciferase assays were performed in HEK293 cells transiently transfected with mouse *Pax6 P1* promoter reporter (pGL4.20 *Pax6 P1*) or with empty vector (pGL4.20), RYBP and RING1 expression plasmids in increasing dosages (*Rybp*: 1 µg, 5 µg, 10 µg, 20 µg; *Ring1*: 1 µg, 10 µg) Values are expressed as fold changes of luciferase activity normalized to the empty vector. The presented values are averages of three independent experiments. Means are standard deviation ± SD. Values of *p* < 0.05 were accepted as significant (*p < 0.05; **p < 0.01; ***p < 0.001, n = 3). Statistical method: *t* test type 3. (**b**) UCSC genome tracks showing ChIP-seq binding of PRC1 factors RING1 (GSM1917303), PCGF2 (GSM1917304), and PRC2 factors SUZ12 (GSM1917301) and EZH2 (GSM1917302)^39^. (**c**) The methylation state of *Pax6* genomic locus in NPCs determined by the histone modifications present using existing ChIP-seq data from Bonev et al., 2017^55^. The binding of RNF2 (GSM2533860), H3K27me3 (GSM2533864), H3K9me3 (GSM2533866), H3K4me1 (GSM2533862), H3K27ac (GSM2533868) and H3K36me3 (GSM2533870) are displayed in reference to CTCF (GSM2533858) and input (GSM2533871). The *Pax6 P1* promoter (highlighted in dotted box) displayed bivalency presenting both repression histone marks (H3K27me3 and H3K9me3) and activation histone mark (H3K27ac) in NPCs. (**d**) The peak density heat map of the genome wide binding targets of RNF2 and PCGF2 integrated with the global position of the CpG islands in NPCs. *Untrans HEK* Untransfected HEK293 cells.

Since RYBP is described for its regulatory functions as a core member of the ncPRC1s, we examined whether RYBP could repress the *Pax6 P1* promoter by a polycomb dependent mechanism. To test this hypothesis, HEK293 cells were co-transfected with the *Pax6 P1* promoter containing luciferase construct (Materials and Methods, Luciferase reporter assay) and constructs expressing RYBP (1 µg, 5 µg, 10 µg and 20 µg) and the catalytic ubiquitin ligase subunit of the ncPRC1 complex, RING1 (1 µg and 10 µg)^38^. We found that the RYBP repression was more pronounced when it was co-transfected with the RING1 as compared to RYBP alone (Fig. 7a). To check whether any members of the PRCs can potentially bind to the *Pax6 P1* promoter, we analyzed existing ChIP-seq data from neural progenitor cells (NPCs)^39^ using UCSC genome browser. Our analysis displayed binding peaks of RNF2, Polycomb group ring finger 2 (PCGF2), SUZ12 Polycomb repressive complex 2 subunit (SUZ12) and Enhancer of Zeste 2 polycomb repressive complex 2 subunit (EZH2) at the *P1* promoter region of *Pax6*. The binding peaks of RNF2 and PCGF2 showed that ncPRC1 complexes could bind to the *Pax6 P1* promoter in NPCs and potentially regulate *Pax6* expression. However, it is likely that the PRC2 complex does not regulate *Pax6* directly, because SUZ12 and EZH2 did not show significant binding (Fig. 7b). The methylation state of the *Pax6* genomic locus in NPCs revealed that, RNF2 peaks coincided with H3K27me3 and H3K9me3 marks showing that the methylation of H3 can potentially play a role in the repression of *Pax6* (Fig. 7c). In ES cells and NPCs the binding peaks of RNF2, CBX factors CBX2, CBX7 and CBX8, which are canonical PRC1 members, revealed a possible dynamic interplay among the PRC1 and ncPRC1 members in maintaining the regulatory status of the *Pax6* genomic locus during differentiation (Fig S3a). Notably, the binding peaks of RNF2, CBX2 and CBX7 factors were present and colocalized at the whole genomic locus suggesting the poised state of the locus. In NPCs, the binding peaks of the same factors were more condensed and specific near the promoter region exhibiting the bivalent state of the promoter, which can be regulated by the presence of specific transcription factors (Fig S3b).

The peak density heatmap showed the global occupancy of ncPRC1s core complex members RNF2 and PCGF2 at the CpG island in NPCs indicating the affinity of the PRC1s binding at the CpG islands. RNF2 binding was more condensed to the position of the CpG islands than PCGF2 suggesting that the position of the CpG islands most likely plays a central role in the repressive activity of ncPRC1s at the *Pax6 P1* promoter, which also includes RYBP (Fig. 7d). These results demonstrated and confirmed further that the RYBP mediated *Pax6* gene repression of its *P1* promoter is polycomb dependent.

## Discussion

In this study we unrevealed that the ncPRC1 core member RYBP is important for proper expression of the genes of the RA signaling pathway, and of their targets, such as *Pax6* during in vitro neural differentiation of mouse ES cells (Figs. 1 and 2). Pax6 is a RA responsive gene and in the *Sey* mice where Pax6 mRNA is expressed at decreased level, the lens anlage cannot respond to exogenous RA after E9.0^40^ confirming that *Pax6* expression was sensitive to RA signaling and that optimal dosage of RA and signaling through RA was required for the development of the embryo proper. RA is a key inductive signal for neural differentiation^41^, optimal RA concentration itself is crucial for appropriate lineage commitment. High concentration of RA induce neural differentiation and low concentration of RA can induce cardiomyocyte differentiation^42^. Based on our current and past studies^23,27^ we think that *Rybp* is important in balancing the proper RA level in cells during differentiation. Thus, *Rybp* might be important for the early steps of differentiation at the time of lineage commitment and tissue formation. Several studies have already shown that *Rybp* is indeed important for lineage commitment. For example, when *Rybp* was depleted from early hematopoietic stem cells and progenitor cells the correct B-1-to-B-2 B-cell progenitor ratio was changed^26^. However, the molecular mechanism of these fate decision making signaling events is still not understood. We have shown previously that the *Rybp*^−/−^ ES cells form less mature neurons, astrocytes, and oligodendrocytes during in vitro neural differentiation while they form neural progenitors abundantly^23^ (Fig S4 and S5). In this study we have shown that *Pax6* expression is elevated during the entire time course of differentiation (at d7, d10 and d14) in the *Rybp*^−/−^ cells while it was silenced properly in the wild type neural cultures. High level of *Pax6* interferes with neural differentiation and can trigger an abundant but developmentally not fully competent neural progenitor pool formation. The *Rybp* heterozygous mutation in mice often led to the overgrowth of midbrain, exencephaly and disorganized, incompletely differentiated neurocortex and the causatives of this phenotype were not clarified yet^22^. The overgrowth of tissues may result from cells with hyperproliferative capacity or even a boost of progenitors, which are trapped at an early phase of differentiation and developmentally compromised. An elevated RA signaling or *Pax6* level can drive accelerated progenitor formation contributing to the heterozygous phenotype and it is reasonable to postulate that increased mRNA expression level of key RA pathway members can alone facilitate *Pax6* expression in the *Rybp* null neural cultures.

By monitoring the relative gene expression changes we have found that the expression of the RA pathway members altered during neural differentiation of *Rybp*^−/−^ ES cells (Fig. 2). These alterations could indirectly lead to more robust expression of several genes, which are regulated by RA, such as *Pax6* and many RA pathway members like *Stra6*, *Crabp1*, *Cyp26a1*^43,44^. The RA inducible trans-membrane receptor *Stra6*, is responsible for the retinol uptake thus high level of *Stra6* (Fig. 2a) in the *Rybp*^−/−^ indicates the possibility of higher retinol uptake of the cells. The elevated expression of *Stra6* alone can cause alterations of RA pathway^45^. Expression of the retinol converting enzyme *Rdh10* (Fig. 2d) showed similar kinetics as *Stra6,* which suggested that an increased amount of RDH10 was required to convert the excess retinol into retinal in the *Rybp*^−/−^ cells. There was elevation in the expression levels of retinal converting enzyme *Aldh1a1* (Fig. 2f) and *Aldh1a2* (Fig. 2g) as well indicating that the conversion of retinal into RA was also possibly enhanced in the *Rybp*^−/−^ cells. After atRA treatment the RA inducible *Crabp1* expression (Fig. 2h) was also upregulated in the *Rybp*^−/−^ cells, suggesting that more RA transported into the nucleus of *Rybp*^−/−^ cells. Finally, another inducible RA pathway member, *Cyp26a1* also showed elevated expression upon atRA treatment in the *Rybp*^−/−^ cells as compared to the wild type cells. All of these effects together can also trigger the accelerated early neural processes and the largely obscured terminal differentiation of neural lineages in the lack of *Rybp*^23^*.*

However, *Rybp* depletion can also cause alteration in PAX6 levels independent to atRA treatment as well (Fig. 3a). Our results showed that the level of PAX6 is elevated (Fig. 3a,c) in the *Rybp*^−/−^ neural cultures even when cells are not yet exposed to atRA. After atRA treatment, the PAX6 protein level remained elevated in the *Rybp*^−/−^ cells (Fig. 4b,c) even at d10 and d14 of in vitro neural differentiation suggesting that RYBP is required for the repression of *Pax6* during neurogenesis (Fig. 5a,b) and the high PAX6 protein level could contribute to the accelerated neural progenitor pool formation that hindered the formation of matured, terminally differentiated neural cultures.

RYBP is a member of the ncPRC1s, which form different complexes based on its PCGF subunit (PCGF1, 2, 3, 4, 5 or 6) they contain^11,17^. The different PCGFs subunits interact with different proteins and regulate different targeting mechanisms and repressor activity. Luciferase reporter assays using *Pax6 P1* promoter (Fig. 6a) confirmed that RYBP could repress the *Pax6* expression (Fig. 6c). E2F and YY1 transcription factors are previously shown to associate with RYBP for the regulation of genes^46^. YY1 is also widely considered to be the DNA binding interactor of ncPRC1s although it is not described to be purified as part of any ncPRC1 complex^17,24^. The presence of TFBSs of several interactors of RYBP and ncPRC1 complexes suggested a possible polycomb mediated repression of the *Pax6 P1* promoter (Fig. 6d). Co-transfection of HEK293 cells with both RYBP and its core polycomb interactor RING1 further promoted the repression of the *Pax6 P1* promoter (Fig. 7a) suggesting that RYBP regulates *Pax6* in a polycomb dependent manner. Polycomb mediated regulation of genes are widely connected to the histone modifications of the given genomic locus^47,48^. The presence of H3K119ub1 and H3K27me3 marks at the gene regulatory regions are both produced and recognized by the PRC1 and PRC2 complexes, respectively^49–51^. Perturbations of PRC1 and PRC2 functions can lead to alterations of different covalent histone modifications^52–54^. Previously published ChIP-seq tracks^55^ of selected histone methylation marks H3K27me3 and H3K9me3 along with RNF2 binding peaks show co-occupancy at the *Pax6* promoter in NPCs (Fig. 7c and Fig S3). Pattern of histone methylation marks can predict the methylation states of the CpG islands present in the regulatory regions of genes^56^. Integrating RNF2 binding peaks and the global position of the CpG islands revealed that RNF2 binding peaks are also condensed at the CpG islands in NPCs (Fig. 7d). The same analysis for PRC2 members SUZ12 and EZH2 exhibited no significant binding but based on the H3K27me3 which is a PRC2 specific methylation mark, it is possible that PRC2 might also function in the repression of *Pax6* (Fig. 7b).

**Figure 8:**
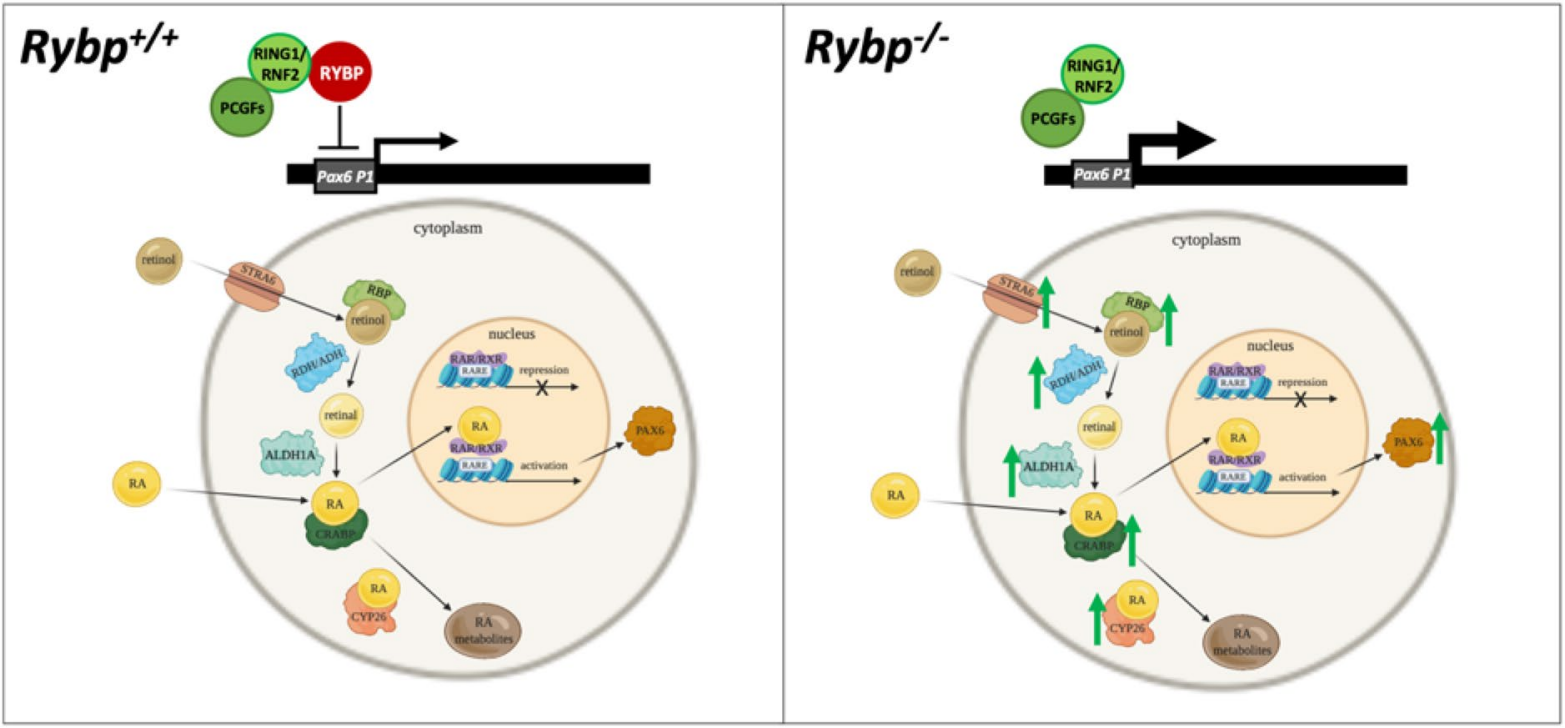
RYBP directs neurogenesis through the regulation of RA pathway and *Pax6*. In the absence of *Rybp* the mRNA expression of the RA pathway members and RYBP target gene *Pax6* are upregulated. Normal *Rybp* level is required to the normal neural differentiation. RYBP can regulate the *Pax6* expression via ncPRC1. The green arrows represent the upregulation of the gene expression of the RA pathway members.

Taken together our results showed that RYBP can repress *Pax6* expression through the *Pax6 P1* promoter and that this repression was further enhanced when RING1 was also present suggesting that RYBP regulates the *Pax6* locus in a polycomb dependent way (Fig. 8). These observations not only contribute to understanding the phenotypic changes of the *Rybp*^−/−^^22,23^, but also to understand the development of neurodegenerative disease conditions when impairment occurs in adult neurogenesis due to abundant progenitor formation^57^.

## Materials and methods

### Cell lines and culture condition

Mouse (129SV/Ola) R1^58^ (mentioned as *Rybp*^+*/*+^ or wild type) and D11^22^ (mentioned as *Rybp*^−/−^ or *Rybp* null mutant) ES cells were cultured as we reported in Henry et al. 2020^27^.

### In vitro neural differentiation

ES cells were induced to differentiate into neuronal lineages as previously described, with some modifications^23,34^. For single-cell suspensions, the cells were dissociated from monolayer culture (day 0) with 0.25% trypsin–EDTA (Trypsin–EDTA (0.5%), Gibco, Cat.No 15400–054). The 8 days old EBs were dissociated and plated and after two days the medium was changed to 1:1 DMEM/F12 (Dulbecco's Modified Eagle Medium/Nutrient Mixture F-12, Gibco, Cat.No 31331-028): Neurobasal medium (Neuronal Base Medium For Neuronal Cells, Gibco, Cat.No 21103-049) with FBS (Foetal Bovine Serum, APS, Cat.No S-001A, USDA grade), 1 mM GlutaMax, 3 mg/ml AlbuMax I, 50U/ml penicillin/streptomycin, 0.5% (vol/vol) N-2 Supplement (N-2 Supplement (100x), Gibco, Cat.No 17502-048), and 1% (vol/vol) B-27 supplement (B-27 Supplement (50x), Gibco, Cat.No 17504-044). Neuroectoderm formation was induced by all-trans retinoic acid (atRA) treatment between day 4 and 8 of differentiation. At day 8 the EBs were collected, trypsinized to single cell suspension, then seeded onto surface coated tissue culture dishes and differentiated further for 6 more days (Fig S2).

### Embryoid body (EB) formation and atRA treatment

Mouse ES cells were harvested as single cell suspension using 0.25% trypsin–EDTA, then seeded at a density of 4.5 × 10^5^ cells/ml in ES medium without LIF into 100 mm diameter bacteriological dishes where cell attachment was prevented. ES cells were kept in suspension for 14 days to form embryoid bodies (EBs). Medium was changed on every second day. EBs were kept in the presence (RA+) and absence (RA-) of 5 μM atRA from the 4th day (d4) of EB formation until the 14th day (d14). The EBs were harvested for ICC analysis on different time points: day 3, 7, 10 and 14 (d3, d7, d10 and d14) (Fig S2).

### Quantitative real-time PCR (qRT-PCR)

RNA was isolated from the cell cultures during the time course of in vitro neural differentiation at the designated time points (d0, d3, d7, d10 and d14) using Gene Jet RNA Purification Kit (Thermo Scientific, Cat.No K0732) according to the manufacturer’s protocol. cDNA synthesis was achieved with the isolated RNA using Applied Biosystems High-capacity cDNA Reverse Transcription Kit (Invitrogen, Cat.No 4368814) according to the manufacturer’s instructions.

qRT-PCR was performed in SYBR Green master mix (SYBR Select Master Mix for CFX, Applied Biosystems, Cat.No 4472942) using Bioer LineGeneK Real-time PCR System (Bioer, China). Relative gene expression changes were quantified using the 2^−ΔΔCt^ method. The threshold cycle (Ct) values for each gene were normalized to the expression level of Hypoxanthine phosphoribosyltransferase 1 (*Hprt*), as internal control. Expression levels were normalized to undifferentiated wild type ES cell samples (d0) which were always set to 1. The primers used in this study were listed at Table S1.

### Immunocytochemistry (ICC)

For immunofluorescence staining ES cells were kept in a single cell suspension culture for d3, d7, d10 and d14 in order to form EBs (see above at Embryoid body (EB) formation and atRA treatment). Samples were collected at different time points (d3, d7, d10, d14) of EB formation and fixed with 4% (v/v) Paraformaldehyde (PFA, Sigma, Cat.No P-6148) for 20 min at room temperature (RT). The EBs and cultured cells were permeabilized with 0.2% Triton X (Triton X-100, Sigma, Cat.No T8787) in PBS (D-PBS (1X) Dulbecco`s Phosphate Buffered Saline, Gibco, Cat. No 14190-094) for 20 min at RT, non-specific binding of the primary antibodies was blocked in 5% BSA (Bovine Serum Albumin, VWR Life science, Cat.No 422361V) in PBS for 1 h at RT. Then EBs (d3–d14) were sequentially incubated with mouse monoclonal PAX6 (Hybridoma Bank, Iowa, USA) and the in vitro neural differentiation samples (d0–d14) were sequentially incubated with mouse monoclonal PAX6 (Hybridoma Bank, Iowa, USA) and rabbit polyclonal RYBP/DEDAF (Merck-Millipore, Cat.No AB3637) primary antibodies. For detection fluorescent-labeled high cross-absorbed secondary antibodies were used (Alexa Fluor 647 Donkey anti Mouse IgG (H + L) (Invitrogen, Cat.No A-31571), Alexa Fluor 488 Donkey anti Rabbit IgG (H + L) (Invitrogen, Cat.No A-21206). For nuclear visualization DAPI (4’,6-diamidino-2-phenylindole, Vector Laboratories, Cat.No H-1200) was used. Stained samples were mounted in Fluoromount-G (Invitrogen, Cat.No 00-4958-02). The images were obtained using Olympus LSM confocal microscope (Olympus Corporation, Japan). Semi-quantification of PAX6 level and the percentage of immune-positive cells were evaluated by ImageJ software with RGB measure method (Rasband, W.S., National Institutes of Health, Bethesda, MD, USA).

### Fluorescence activated flow cytometry analysis (FACS)

Cells were collected at d0, d4, d6, d7, d8, d10 and d14 from in vitro neural differentiation and fixed with 70% ice cold ethanol for 20 min at RT. Next cells were washed with washing solution (PBS with 0.5% BSA). For denaturation 2 M HCl was used for 10 min at RT. After washing the neutralization of the residual acid was done with 0.1 M sodium borate (pH 8.5). Cells were washed and single or double stained with mouse monoclonal PAX6 (Hybridoma Bank, Iowa, USA) and rabbit polyclonal RYBP/DEDAF (Merck-Millipore, Cat.No AB3637) primary antibodies for 30 min at RT followed with Alexa Fluor 647 labelled Donkey anti Mouse IgG (H + L) (Invitrogen, Cat.No A-31571) and Alexa Fluor 488 labelled Donkey anti Rabbit IgG (H + L) (Invitrogen, Cat.No A-21206), respectively for 20 min at RT. All antibodies were diluted in dilution solution (washing solution containing 0.5% BSA and 0.5% TWEEN-20 (Sigma-Aldrich, Cat.No: P5927) according to the manufacturers. For negative controls, first antibodies were omitted and cells were labelled only with secondary antibodies at each examined time points.

We measured the fluorescence of 50,000 cells/sample with Becton Dickinson FACS Calibur fluorescent flow cytometer with 488 nm and 633 nm laser for Alexa Fluor 488 and Alexa Fluor 647 labelled secondary antibodies, respectively. Data were analyzed by BD CellQuest Pro Version 6.0 software.

### Metadata analysis of previously published RNA-seq and ChIP-seq datasets

Transcriptome analysis of wild type and *Rybp*^−/−^ ES cells RNA-seq dataset was previously published^30^ (GEO acc. GSE151349). RColorBrewer and pheatmap packages in R were used to generate heatmap.

ChIP-seq datasets of H2AK119ub1 in wild type and *Rybp* KO ES cells were previously published^33^ (SRA acc. SRP247114). Processed ChIP-seq datasets were visualized as BedGraph files in Integrative Genomics Viewer (IGV)^59^.

ChIP-seq datasets of PRC1 factors RING1 (GSM1917303), PCGF2 (GSM1917304), and PRC2 factors SUZ12 (GSM1917301), EZH2 (GSM1917302) were analyzed in UCSC genome browser. The methylation state of *Pax6* genomic locus in NPCs were analyzed from RNF2 (GSM2533860), H3K27me3 (GSM2533864), H3K9me3 (GSM2533866), H3K4me1 (GSM2533862), H3K27ac (GSM2533868) and H3K36me3 (GSM2533870) were visualized in reference to CTCF (GSM2533858) and input (GSM2533871) in IGV tracks.

ChIP-seq datasets from GSE89929 were analyzed as BedGraph files in IGV to compare the binding of PRC1 factors in ES cells and NPC’s.

### Cloning of *Pax6 P1* promoter

*Pax6 P1* promoter was PCR amplified from *Pax6* BAC clone (RP23-259M2) using specific primers 5’—ATATGGTACCTTTATTGTCAATCTCTGTCTT—3’ (introduces KpnI site at 5’ end) and 5’—ACGTGCTAGCCTATCTAATTACCTAAGTA—3’ (introduces NheI site at 3’). This PCR fragment was digested overnight with KpnI (New England BioLabs, Cat.No: R0142L) and NheI (New England BioLabs, Cat.No: R3131L) and cloned into the same sites of pGL4.20 (pGL4.20 (*luc2*/Puro), Promega, Cat.No E6751) luciferase containing vector.

### Luciferase reporter assay

HEK293 cells were seeded at a density of 8 × 10^5^ cells per 6 cm plates and transiently co-transfected with the following vectors: pGL4-*Pax6*-Luc, pcDNA3.1-*Rybp*-HA^35^ and pcDNA3.1-*Ring1*-FLAG. The HEK293 cells were transfected these vectors in various concentrations (pGL4-*Pax6*-Luc: 5 µg; pcDNA3.1-*Rybp*-HA: 1 µg, 5 µg, 10 µg and 20 µg; pcDNA3.1-*Ring1*-FLAG: 1 µg and 10 µg) using CaPO_4_ transfection method^60,61^. CaPO_4_ transfection were performed as follows: 8 h before transfection the cell culture medium was changed, the vectors were diluted in TE (Tris–EDTA buffer solution, Sigma, Cat.No 93283) buffer, then 2.5 M CaCl_2_ and 2 × HBS (Hepes Buffered Saline, Sigma, Cat.No H3375) were added in dropwise to the solution while the mix was bubbled to providing oxygenation, finally this solution was added to the medium. 16 h after transfection, the cell culture medium was changed. 48 h after transfection the cells were washed with PBS and lysed with 1 × Passive lysis buffer provided by the Luciferase reporter assay kit (Dual Luciferase Reporter Assay System, Promega, Cat.No E1500). 20 µg of lysate from each point was mixed with 100 µl Luciferase Assay Reagent II substrate (provided by the kit) and the induced luciferase activity was recorded immediately according to the manufacturer’s instructions. The luciferase activity was recorded with Perkin Elmer TopCount NXT Luminometer. All activities were measured in triplicates.

### Statistical analysis

All experiments were repeated three times from three independent biological samples (by defrosting independent vial of cells) and technical repeats were used as triplicates (e.g. three petri dishes/sampeling) at each examined timepoints. Experiments were evaluated with Microsoft Excel by using Student T-test type 3. Means are standard deviation. Values of *p* < 0.05 were accepted as significant (*p < 0.05; **p < 0.01; ***p < 0.001).

## Supplementary Information

Supplementary Information.

## References

[1] Chuang J, Tung L, Lin Y (2015). Neural differentiation from embryonic stem cells in vitro: An overview of the signaling pathways. World J. Stem Cells.

[2] Tonge PD, Andrews PW (2010). Retinoic acid directs neuronal differentiation of human pluripotent stem cell lines in a non-cell-autonomous manner. Differ. Res. Biol. Divers..

[3] Bonney S (2016). Diverse functions of retinoic acid in brain vascular development. J. Neurosci..

[4] Gajović S, St-Onge L, Yokota Y, Gruss P (1997). Retinoic acid mediates Pax6 expression during in vitro differentiation of embryonic stem cells. Differ. Res. Biol. Divers..

[5] Rhinn M, Dollé P (2012). Retinoic acid signalling during development. Development.

[6] Thompson B (2021). Overview of PAX gene family: analysis of human tissue-specific variant expression and involvement in human disease. Hum. Genet..

[7] Zhang X (2010). Pax6 is a human neuroectoderm cell fate determinant. Cell Stem Cell.

[8] Ericson J (1997). Pax6 controls progenitor cell identity and neuronal fate in response to graded Shh signaling. Cell.

[9] Margueron R, Reinberg D (2011). The Polycomb complex PRC2 and its mark in life. Nature.

[10] Shaver S, Casas-Mollano J, Cerny R, Cerutti H (2010). Origin of the polycomb repressive complex 2 and gene silencing by an E(z) homolog in the unicellular alga Chlamydomonas. Epigenetics.

[11] Gao Z (2012). PCGF homologs, CBX proteins, and RYBP define functionally distinct PRC1 family complexes. Mol. Cell.

[12] Luis N, Morey L, Di Croce L, Benitah S (2012). Polycomb in stem cells: PRC1 branches out. Cell Stem Cell.

[13] Morey L (2012). Nonoverlapping functions of the Polycomb group Cbx family of proteins in embryonic stem cells. Cell Stem Cell.

[14] O’Loghlen A (2012). MicroRNA regulation of Cbx7 mediates a switch of Polycomb orthologs during ESC differentiation. Cell Stem Cell.

[15] Tavares L (2012). RYBP-PRC1 complexes mediate H2A ubiquitylation at polycomb target sites independently of PRC2 and H3K27me3. Cell.

[16] Morey L, Aloia L, Cozzuto L, Benitah S, Di Croce L (2013). RYBP and Cbx7 define specific biological functions of polycomb complexes in mouse embryonic stem cells. Cell Rep..

[17] Bajusz, I., Kovács, G. & Pirity, M. K. From flies to mice: the emerging role of non-canonical PRC1 members in mammalian development. *Epigenomes 2*, 4 (2018).

[18] Fukuda T, Tokunaga A, Sakamoto R, Yoshida N (2011). Fbxl10/Kdm2b deficiency accelerates neural progenitor cell death and leads to exencephaly. Mol. Cell. Neurosci..

[19] Hao Y (2015). USP7 acts as a molecular rheostat to promote WASH-dependent endosomal protein recycling and is mutated in a human neurodevelopmental disorder. Mol. Cell.

[20] Tang Y (2017). Protein deubiquitinase USP7 is required for osteogenic differentiation of human adipose-derived stem cells. Stem Cell Res. Ther..

[21] Almeida M (2017). PCGF3/5-PRC1 initiates Polycomb recruitment in X chromosome inactivation. Science.

[22] Pirity MK, Locker J, Schreiber-Agus N (2005). Rybp/DEDAF Is required for early postimplantation and for central nervous system development. Mol. Cell. Biol..

[23] Kovacs, G., Szabo, V. & Pirity, M. K. Absence of rybp compromises neural differentiation of embryonic stem cells. *Stem Cells Int.* **2016**, (2016). 10.1155/2016/4034620 PMC4693026 26788067

[24] Garcia, E., Marcos-Gutiérrez, C., Del Mar Lorente, M., Moreno, J. C. & Vidal, M. RYBP, a new repressor protein that interacts with components of the mammalian Polycomb complex, and with the transcription factor YY1. *EMBO J.* **18**, 3404–3418 (1999). 10.1093/emboj/18.12.3404 PMC1171420 10369680

[25] Tian Q, Guo S-M, Xie S-M, Yin Y, Zhou L-Q (2020). Rybp orchestrates spermatogenesis via regulating meiosis and sperm motility in mice. Cell Cycle Georget. Tex.

[26] Calés C (2016). Role of Polycomb RYBP in maintaining the B-1-to-B-2 B-cell lineage switch in adult hematopoiesis. Mol. Cell. Biol..

[27] Henry S, Szabó V, Sutus E, Pirity MK (2020). RYBP is important for cardiac progenitor cell development and sarcomere formation. PLoS ONE.

[28] Wang W (2014). RYBP expression is associated with better survival of patients with hepatocellular carcinoma (HCC) and responsiveness to chemotherapy of HCC cells in vitro and in vivo. Oncotarget.

[29] Zheng L, Schickling O, Peter ME, Lenardo MJ (2001). The death effector domain-associated factor plays distinct regulatory roles in the nucleus and cytoplasm. J. Biol. Chem..

[30] Ujhelly, O. *et al.* Lack of Rybp in mouse embryonic stem cells impairs cardiac differentiation. *Stem Cells Dev.* **24**, (2015). 10.1089/scd.2014.0569 26110923

[31] Balmer J, Blomhoff R (2002). Gene expression regulation by retinoic acid. J. Lipid Res..

[32] Thompson, B. *et al.* Genetics and functions of the retinoic acid pathway, with special emphasis on the eye. *Hum. Genom. 2019 131* **13**, 1–15 (2019). 10.1186/s40246-019-0248-9 PMC6892198 31796115

[33] Zhao J (2020). RYBP/YAF2-PRC1 complexes and histone H1-dependent chromatin compaction mediate propagation of H2AK119ub1 during cell division. Nat. Cell Biol..

[34] Bibel M (2004). Differentiation of mouse embryonic stem cells into a defined neuronal lineage. Nat. Neurosci..

[35] Arrigoni R (2006). The Polycomb-associated protein Rybp is a ubiquitin binding protein. FEBS Lett..

[36] Krätzner R (2008). A peroxisome proliferator-activated receptor γ-retinoid X receptor heterodimer physically interacts with the transcriptional activator PAX6 to inhibit glucagon gene transcription. Mol. Pharmacol..

[37] Aota SI (2003). Pax6 autoregulation mediated by direct interaction of Pax6 protein with the head surface ectoderm-specific enhancer of the mouse Pax6 gene. Dev. Biol..

[38] Pirity MK (2007). Rybp, a polycomb complex-associated protein, is required for mouse eye development. BMC Dev. Biol..

[39] Kloet SL (2016). The dynamic interactome and genomic targets of Polycomb complexes during stem-cell differentiation. Nat. Struct. Mol. Biol..

[40] Enwright JF, Grainger RM (2000). Altered retinoid signaling in the heads of small eye mouse embryos. Dev. Biol..

[41] Lu J (2009). All-trans retinoic acid promotes neural lineage entry by pluripotent embryonic stem cells via multiple pathways. BMC Cell Biol..

[42] Rohwedel J, Guan K, Wobus AM (1999). Induction of cellular differentiation by retinoic acid in vitro. Cells Tissues Organs.

[43] Mangelsdorf DJ (1991). A direct repeat in the cellular retinol-binding protein type II gene confers differential regulation by RXR and RAR. Cell.

[44] Piedrafita, F. J. & Pfahl, M. Nuclear Retinoid Receptors and Mechanisms of Action. *Nau H Blaner WS Eds Retin. Handb. Exp. Pharmacol.* **139**, 153–184 (1999).

[45] Wu L, Ross A (2010). Acidic retinoids synergize with vitamin A to enhance retinol uptake and STRA6, LRAT, and CYP26B1 expression in neonatal lung. J. Lipid Res..

[46] Schlisio S, Halperin T, Vidal M, Nevins JR (2002). Interaction of YY1 with E2Fs, mediated by RYBP, provides a mechanism for specificity of E2F function. EMBO J..

[47] Boyer LA (2006). Polycomb complexes repress developmental regulators in murine embryonic stem cells. Nature.

[48] Blackledge NP (2020). PRC1 catalytic activity is central to polycomb system function. Mol. Cell.

[49] Cao R (2002). Role of histone H3 lysine 27 methylation in Polycomb-group silencing. Science.

[50] Müller J (2002). Histone methyltransferase activity of a Drosophila Polycomb group repressor complex. Cell.

[51] Issa J-P (2011). Epigenetics. FEBS Lett..

[52] Endoh M (2008). Polycomb group proteins Ring1A/B are functionally linked to the core transcriptional regulatory circuitry to maintain ES cell identity. Development.

[53] Rose, N. R. *et al.* RYBP stimulates PRC1 to shape chromatin-based communication between polycomb repressive complexes. *eLife* **5**, 1–29 (2016). 10.7554/eLife.18591 PMC5065315 27705745

[54] Fursova NA (2019). Synergy between variant PRC1 complexes defines polycomb-mediated gene repression. Mol. Cell.

[55] Bonev B (2017). Multiscale 3D genome rewiring during mouse neural development. Cell.

[56] Fan S, Zhang M, Zhang X (2008). Histone methylation marks play important roles in predicting the methylation status of CpG islands. Biochem. Biophys. Res. Commun..

[57] Bender, H., Fietz, S. A., Richter, F. & Stanojlovic, M. Alpha-synuclein pathology coincides with increased number of early stage neural progenitors in the adult hippocampus. *Front. Cell Dev. Biol.* **0**, 1802 (2021). 10.3389/fcell.2021.691560 PMC8293917 34307368

[58] Nagy A, Rossant J, Nagy R, Abramow-Newerly W, Roder JC (1993). Derivation of completely cell culture-derived mice from early-passage embryonic stem cells. Proc. Natl. Acad. Sci. USA.

[59] Robinson J, Thorvaldsdóttir H, Wenger A, Zehir A, Mesirov J (2017). Variant review with the integrative genomics viewer. Cancer Res..

[60] Wigler M (1979). Transformation of mammalian cells with genes from procaryotes and eucaryotes. Cell.

[61] Kingston, R. E., Chen, C. A. & Rose, J. K. Calcium phosphate transfection. *Curr. Protoc. Mol. Biol.* **63**, 9.1.1–9.1.11 (2003). 10.1002/0471142727.mb0901s63 18265332

