# Supplementary material for: RYBP regulates Pax6 during in vitro neural differentiation of mouse embryonic stem cells
Source: Sci Rep. 2022 Feb 11;12:2364. doi: 10.1038/s41598-022-06228-1 (PMC8837790; doi:10.1038/s41598-022-06228-1)
Supplement: Supplementary file 1 — Supplementary Information. [file 41598_2022_6228_MOESM1_ESM.docx]

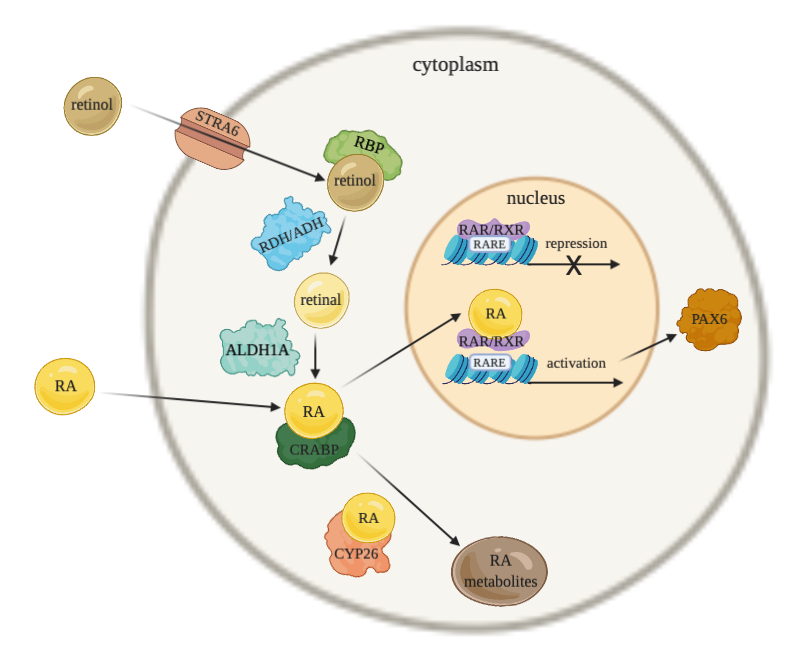


**Figure S1 – The schematic illustration of the RA signaling pathway in cells**

Retinol enters the target cells through the Signaling receptor and transporter of retinol (STRA6) and is stabilized by the Cellular retinol binding proteins (RBPs) within the cells. The Retinol dehydrogenases (RDHs) converts retinol into retinal and the activity of Aldehyde dehydrogenase 1 family member A (ALDH1As) is needed to produce RA, while Cytochrome P450 family (CYP26) degrades the excess RA. Cellular retinoic acid binding proteins (CRABPs) transport RA into the nucleus. In the nucleus the RA binds to the RA receptor and Retinoid X receptor (RAR/RXR) heterodimers, which binds to RA response elements (RAREs) in the regulatory region of RA target genes and initiates transcription.


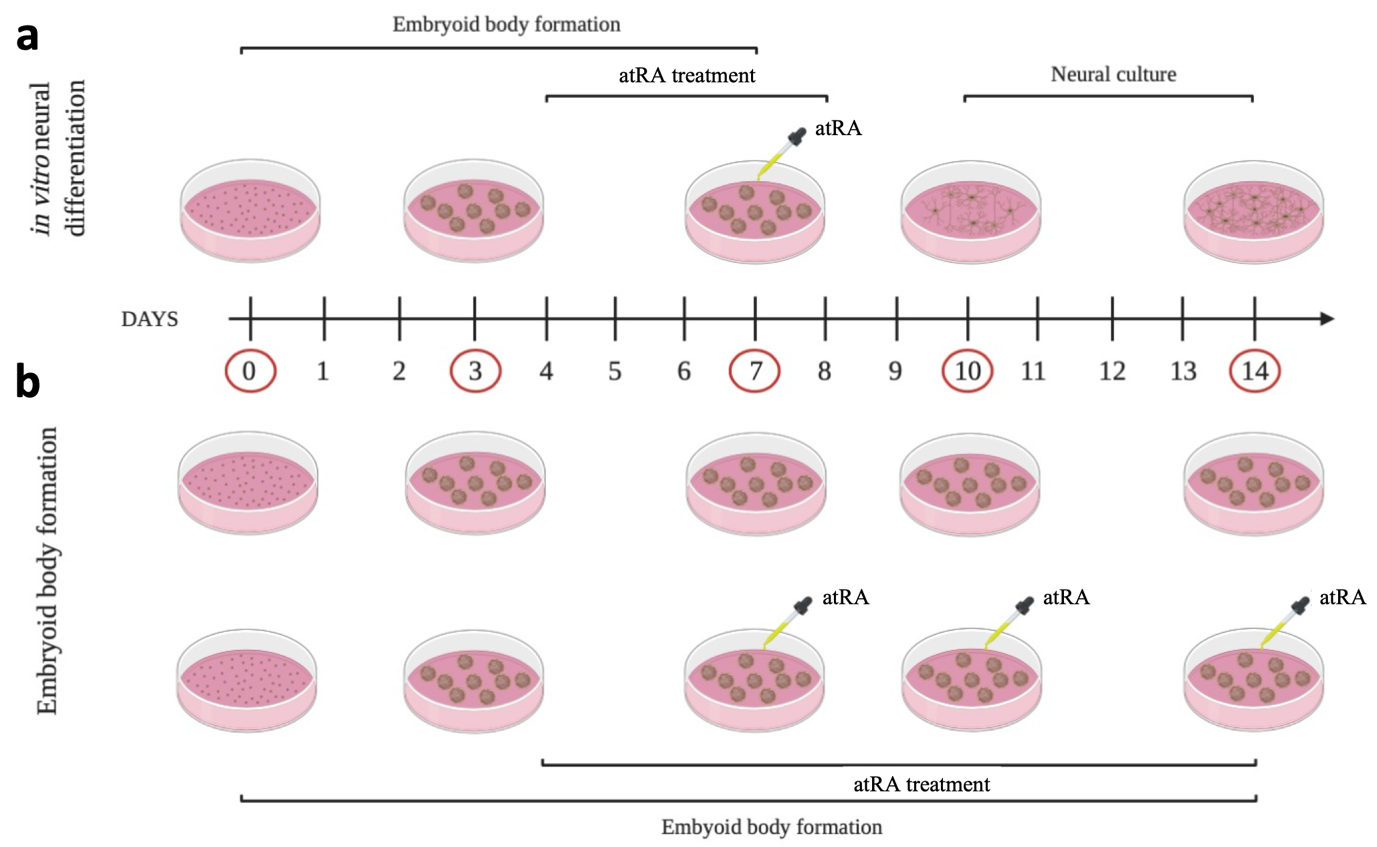


**Figure S2** – (**a**) Schematic illustration of *in vitro* neural differentiation as described by Bibel et al.^23^ (**b**) Schematic illustration of embryoid body (EB) formation in the presence and the absence of atRA. Time points of sample collection are indicated with red circles.


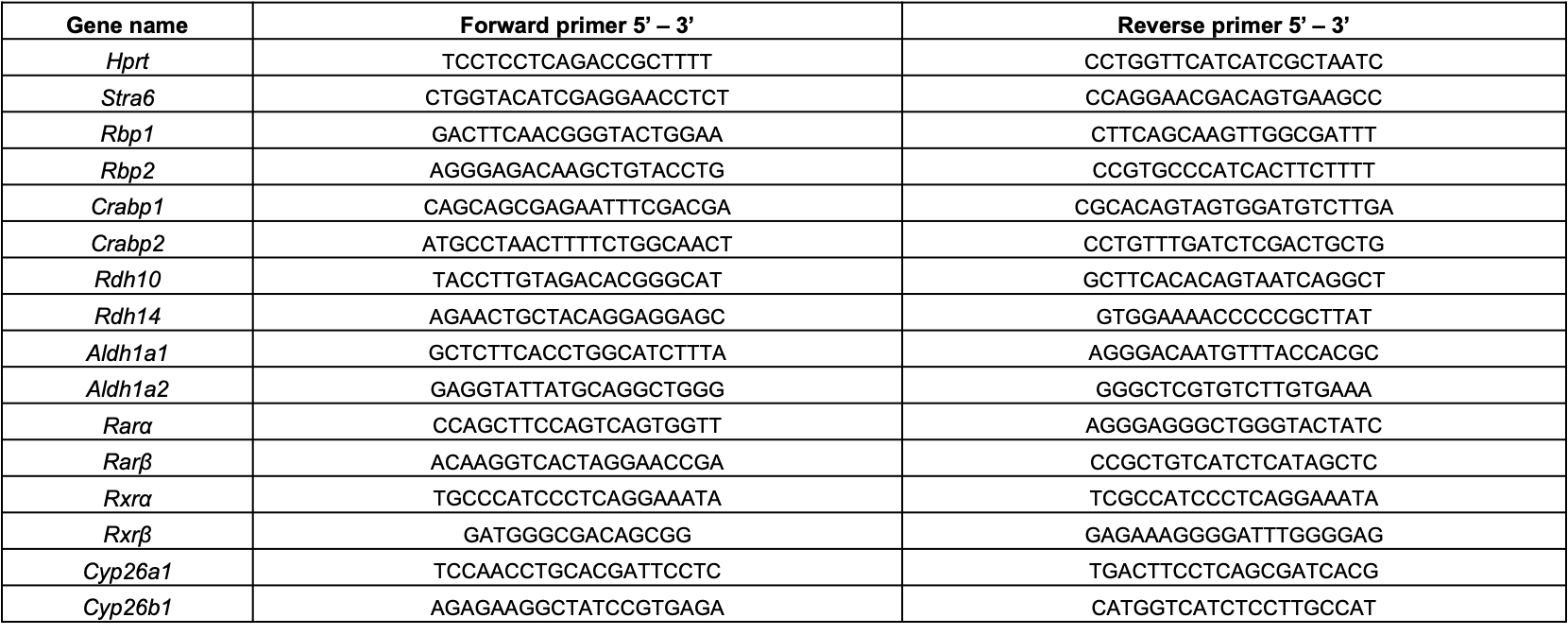


**Table S1** - Primers used in qRT-PCR reactions

******

**Figure S3** – The binding peaks of RYBP (GSM4052119), RNF2 (ES: GSM2393579, NPC: GSM2393586), Chromobox (CBX) factors CBX2 (ES: GSM2393582, NPC: GSM2393588), CBX7 (GSM2393581), CBX8 (GSM2393587), H3K27me3 (ES: GSM2393585, NPC: GSM2393591) and H2AK119ub1 (ES: GSM2393583, NPC: GSM2393589) at the *Pax6* genomic locus in **(a)** ES cells and **(b)** NPCs.

**Figure S4** - (**a**) Immunocytochemical analysis of neural progenitor marker PAX6 and late neural marker β-III-tubulin visualized by anti-TUJ1 antibody. Objective: 40x. Scale bar: 80 μm. (**b**) Immunocytochemical analysis of PAX6 and TUJ1 protein level. Objective: d3, d7: 40x, d10, d14: 60x. Scale bar: d3, d7: 80 μm, d10,d14: 1000 μm. (**c, d** ) ImageJ quantification of PAX6 protein level in the wild type and *Rybp* null mutant cells during the *in vitro* neural differentiation in the presence (D) and absence (C) of atRA. Means are standard deviation ± SD. Values of p ˂ 0.05 were accepted as significant (* p < 0.05; ** p < 0.01; *** p < 0.001). Statistical method: T test type 3, n=2.

**Figure S5** - (**a**) Immunocytochemical analysis of RYBP and radial glial marker RC2. Objective: 40x. Scale bar: 80 μm. (**b**) Immunocytochemical analysis of RYBP and RC2 protein level. Objective: d3, d7: 40x, d10, d14: 60x. Scale bar: d3, d7: 80 μm, d10,d14: 1000 μm. (**c, d** ) ImageJ quantification of PAX6 protein level in the wild type ad *Rybp* null mutant cells during the *in vitro* neural differentiation in the presence (D) and absence (C) of atRA. Means are standard deviation ± SD. Values of p ˂ 0.05 were accepted as significant (* p < 0.05; ** p < 0.01; *** p < 0.001). Statistical method: T test type 3, n=2.
